# Characterization of two novel *Salmonella* phages having biocontrol potential against *Salmonella* spp. in gastrointestinal conditions

**DOI:** 10.1038/s41598-024-59502-9

**Published:** 2024-05-29

**Authors:** Yujie Zhang, Mackenna Chu, Yen-Te Liao, Alexandra Salvador, Vivian C. H. Wu

**Affiliations:** Produce Safety and Microbiology Research Unit, U.S. Department of Agriculture, Agricultural Research Service, Western Regional Research Center, 800 Buchanan Street, Albany, CA 94710 USA

**Keywords:** Microbiology, Bacteriophages

## Abstract

*Salmonella* is a primary enteric pathogen related to the contamination of poultry and other food products in numerous foodborne outbreaks. The continuous emergence of multidrug-resistant bacteria has become a serious issue due to the overuse of antibiotics. Hence, lytic phages are considered alternative biocontrol agents against these bacterial superbugs. Here, two *Salmonella* phages—S4lw and D5lw—were subjected to genomic and biological characterization and further encapsulated to improve the stability under acidic conditions mimicking gastrointestinal conditions. The two lytic phages, S4lw and D5lw, taxonomically belong to new species under the *Guernseyvirinae* and *Ackermannviridae* families, respectively. Each phage showed antimicrobial activities against diverse *Salmonella* spp., such as *S.* Enteritidis and *S.* Typhimurium, achieving 1.7–3.4 log reduction after 2–6 h of treatment. The phage cocktail at a multiplicity of infection (MOI) of 100 or 1000 completely inhibited these *Salmonella* strains for at least 14 h at 25 °C. Additionally, the bead-encapsulated phage cocktail could withstand low pH and different simulated gut environments for at least 1 h. Overall, the newly isolated phages can potentially mitigate *Salmonella* spp. under the gastrointestinal environments through encapsulation and may be further applied via oral administration to resolve common antimicrobial resistance issues in the poultry production chain.

## Introduction

The poultry industry has grown drastically worldwide due to the increasing demand for the consumption of poultry meat and other related food products. The profit of low-cost production and high consumer demand have greatly driven the industry to produce nearly 100 million tons of poultry food products per year^1^. However, the pervasiveness of several common pathogens, including *Salmonella, Listeria*, and *Campylobacter*, has become a severe concern in the poultry industry^2^. In particular, *Salmonella* is widely detected in the poultry gastrointestinal tract, posing potential food safety risks to human health, such as Salmonellosis^3^. Two serovars of *Salmonella* from poultry origins were the primary cause of the human infection, of which 26% were of *Salmonella enterica* Enteritidis (*S.* Enteritidis), and 22% were of *Salmonella enterica* Typhimurium (*S.* Typhimurium)^4^. *Salmonella* infections can be elicited by direct human-to-poultry interaction, contamination of animal waste, and, most importantly, contaminated food. The continuous emergence of foodborne pathogens and their cross-contamination of food products during the processing pipeline in poultry facilities could result in a serious issue that requires extra effort.

Although *Salmonella* is one of the preeminent agents constantly contributing to foodborne illnesses in the United States, its capability to develop antibiotic resistance renders a greater threat to public health. The overuse and misuse of antibiotics in poultry have been the leading factors contributing to the increased antibiotic resistance in livestock. It has been reported that antibiotic-resistant *Salmonella* strains were commonly isolated from chicken meat around the world^5^. In the poultry production chain worldwide, such as poultry farms, layer farms, chickens in processing plants, and supermarkets, the presence of *Salmonella* was detected, with the highest resistance levels to nalidixic acid and ampicillin^5^. Moreover, due to the prevalence of antibiotic-resistant *Salmonella*, children, seniors, and immunosuppressed populations are much more susceptible to fatal infections^6^. The inability to use antibiotics as a standard treatment for those with Salmonellosis presents a future food safety threat to the public.

Bacteriophages (or phages) are viruses that infect bacteria. Based on the phage lifecycle, lytic phages can lyse their target bacterial host as natural predators. Therefore, lytic bacteriophages have been a promising antimicrobial agent for combating bacterial pathogens in the food industry. For example, several commercial phage products against foodborne pathogens—*Listeria monocytogenes*, *Salmonella* spp., and *E. coli* O157:H7—have been approved for application in food or production environments^7^. The ability of phages to explicitly target specific bacteria without harming the background microflora and host animal has been a highly desirable approach to treating *Salmonella* contamination. Additionally, the combination of diverse phages was vastly effective in eliminating target bacteria compared to individual phages. Several studies have investigated the antimicrobial activity of phage cocktails against *Salmonella *in vitro and in vivo. A previous study used a phage cocktail (CNPSA1, CNPSA3, and CNPSA4) to treat broiler chickens infected with *S.* Enteritidis PT4; phage-treated chickens had a 3.5-fold reduction in *Salmonella* cecal count compared to the control group^8^. In addition, several studies have focused on phage oral administration in chickens to improve the treatment effectiveness^9,10^. In most cases, phages were added to animal feeds or drinking water to enable effective oral administration into the chicken GI tract to control bacterial pathogens. One study indicated that a decreasing mortality rate of chickens and the absence of *S.* Typhimurium and *S.* Enteritidis in the chick cecum were observed after five successive doses of orally administered phages^11^. Most of all, using phages in chicken feed and water did not alter the chicken’s health or behavior, ensuring that phages are not toxic to host organisms nor affect their characteristics^12^. To diversify the library of *Salmonella* phages, this study characterized two newly isolated *Salmonella* phages—S4lw and D5lw—using genomic and biological approaches. In addition, the encapsulation of a two-phage cocktail (S4lw + D5lw) was further conducted to estimate their stability to sustain acidic stress in different simulated poultry gastrointestinal environments for controlling *Salmonella* contamination via oral administration.

## Results

### Phage genomic features

**Figure 1:**
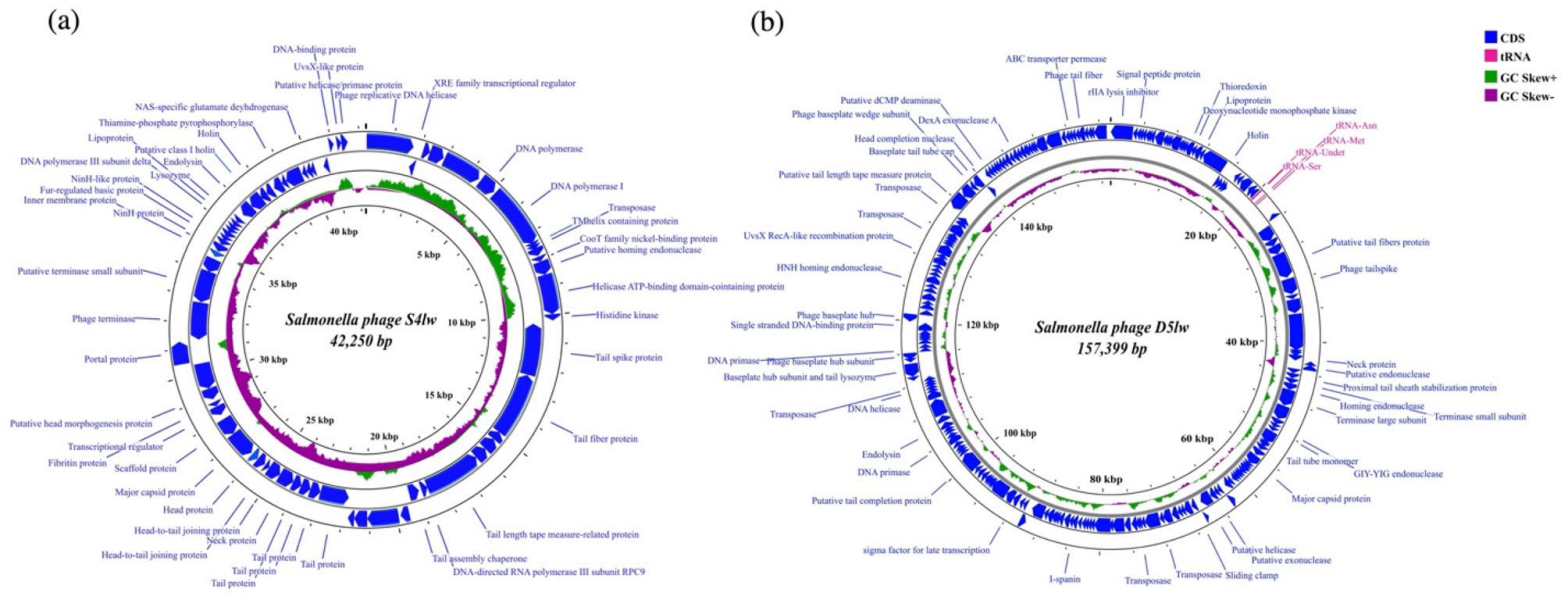
Circular genome maps of phages S4lw (**a**) and D5lw (**b**) generated using CGview server^BETA^. The rings from the inside out represent GC skew (green and purple), CDSs (dark blue), and tRNAs (red). tRNAs are only detected in phage D5lw (**b**).

**Figure 2:**
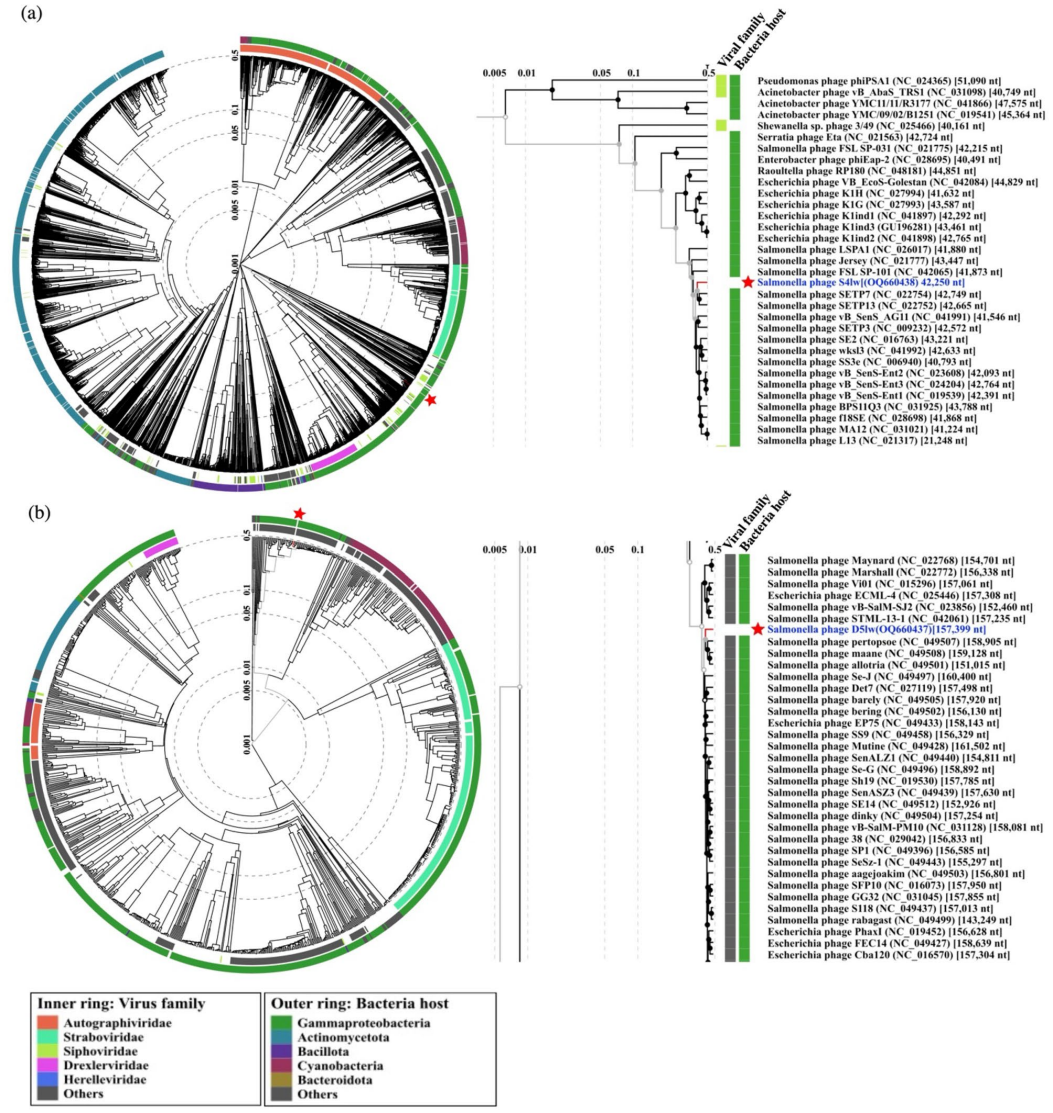
Phylogenetic analysis of phages S4lw (**a**) and D5lw (**b**) constructed with the closely related phages using ViPTree database based on the alignment of phage complete genomes. The red stars indicate where phages S4lw and D5lw are positioned outside the trees for easy visualization. A zoom-in phylogenic tree is presented on the right panel of each phage containing the most closely related phage genomes to S4lw or D5lw. The viral family and bacterial host phylum classified by ViPTree are shown in the figure legend.

**Figure 3:**
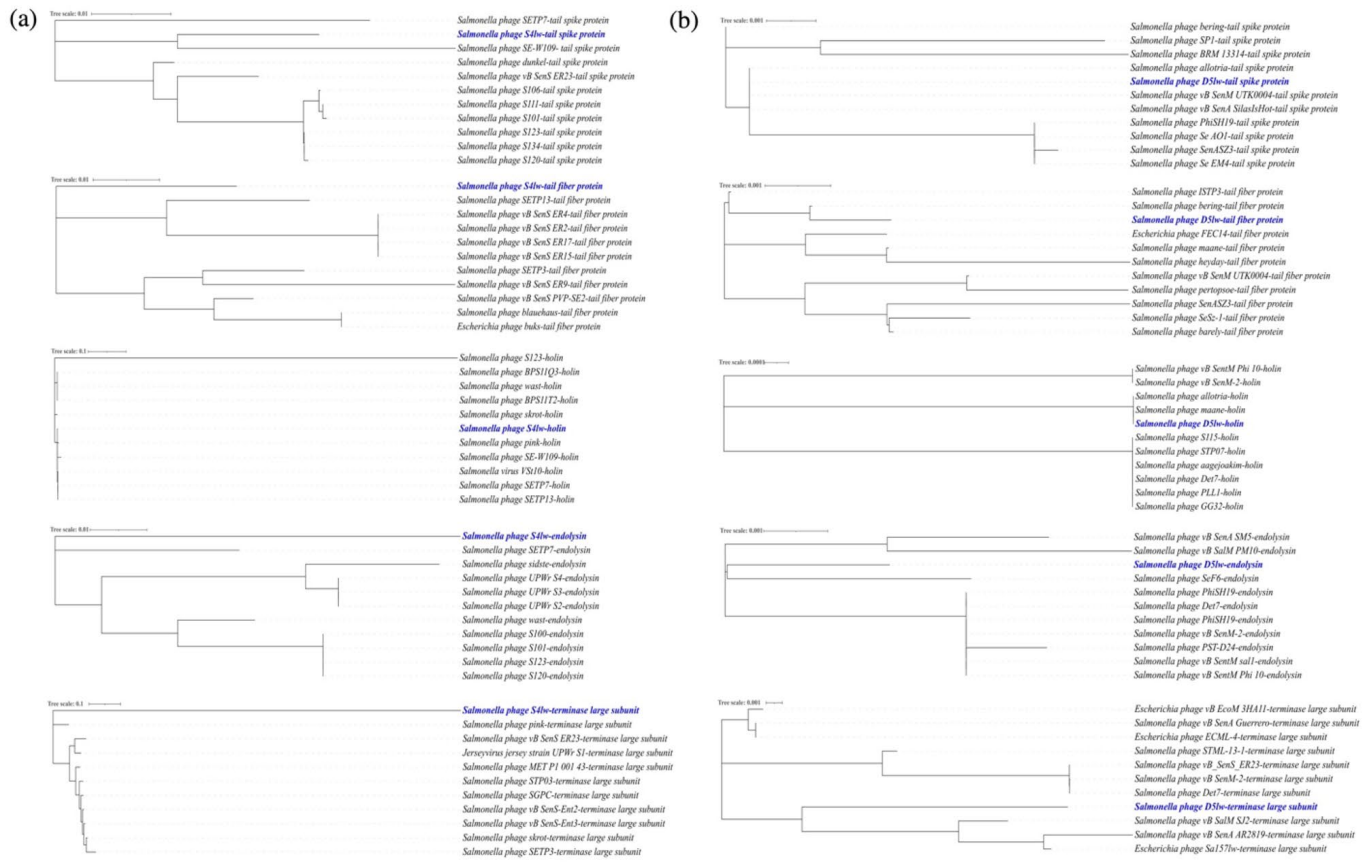
Maximum likelihood phylogenetic analysis of phages S4lw (**a**) and D5lw (**b**) with the closely related reference phages based on the ClustalW alignment of the CDSs associated with phage infection (tail spike and tail fiber), host cell lysis (holin and endolysin), and DNA packaging (terminase).

Phage S4lw contained double-stranded DNA with a 42,250-bp genome size and an average GC content of 49.7%. Sixty-nine coding DNA sequences (CDSs) were annotated in the genome of S4lw, 50 of which were predicted with the functions related to phage structure, DNA replication and repair, and host bacterial recognition and lysis (Fig. 1a). No virulence, antibiotic resistance, or lysogenic genes were detected in this phage genome. In addition, the genetic taxonomy results indicated that phage S4lw belonged to the *Jerseyvirus* genus under the *Guernseyvirinae* family. The phylogenetic tree showed that phage S4lw shared a close evolutionary relationship with *Salmonella* phage SETP7 (NC_022754) and *Salmonella* phage SETP13 (NC_022752) (Fig. 2a). In addition, the CDSs related to the crucial phage biological features, including bacterial host recognition (tail fiber, tail spike), host cell lysis (holin, endolysin), and phage replication (terminase large subunit), were genomically compared with the counterpart of other reference phages (Fig. 3a). The comparative genomics revealed that phage S4lw contained unique functional CDSs coding for tail fiber protein, holin, endolysin, and the terminase large subunit compared to the reference phages. Only the CDS encoding for tail spike protein in the phage S4lw shared a high nucleotide sequence similarity with the counterpart of *Salmonella* phage SE-W109.

Phage D5lw had double-stranded DNA with a genome size of 157,399-bp and an average GC content of 44.7%. A total of 208 coding DNA sequences (CDSs) and 4 tRNAs (tRNA-Undet, tRNA-Ser, tRNA-Asn, and tRNA-Met) were found in the phage genome (Fig. 1b). There was no presence of harmful genes, such as virulence, antibiotic resistance, or lysogenic genes, in the phage D5lw genome. The genetic taxonomy indicated that phage D5lw was classified in the *Kuttervirus* genus under the *Ackermannviridae* family. D5lw was genomically similar to three *Salmonella* phages: Pertopsoe (NC_049507), Maane (NC_049508) and Allotira (NC_049501) (Fig. 2b). The CDSs related to critical biological traits were further compared with the counterparts of the close-related reference phages, as shown in Fig. 3b. The predicted CDSs coding for tail spike and tail fiber proteins in phage D5lw, related to phage infection, were closely related to that of *Salmonella* phage vB SenM UTK0004 and *Salmonella* phage Bering, respectively. The CDSs of lysin and holin, both associated with host lysis, in the D5lw genome showed a high nucleotide similarity to the counterparts in *Salmonella* phage Maane and *Salmonella* phage D3F6, respectively (Fig. 3b). Furthermore, phage D5lw contained the CDS of terminase large subunit, sharing a high nucleotide sequence similarity with *Salmonella* phage vB SalM SJ2, *Salmonella* phage vB SenA AR2819 and *Escherichia* phage Sa157lw (Fig. 3b).

### Biological characterization of phages

**Figure 4:**
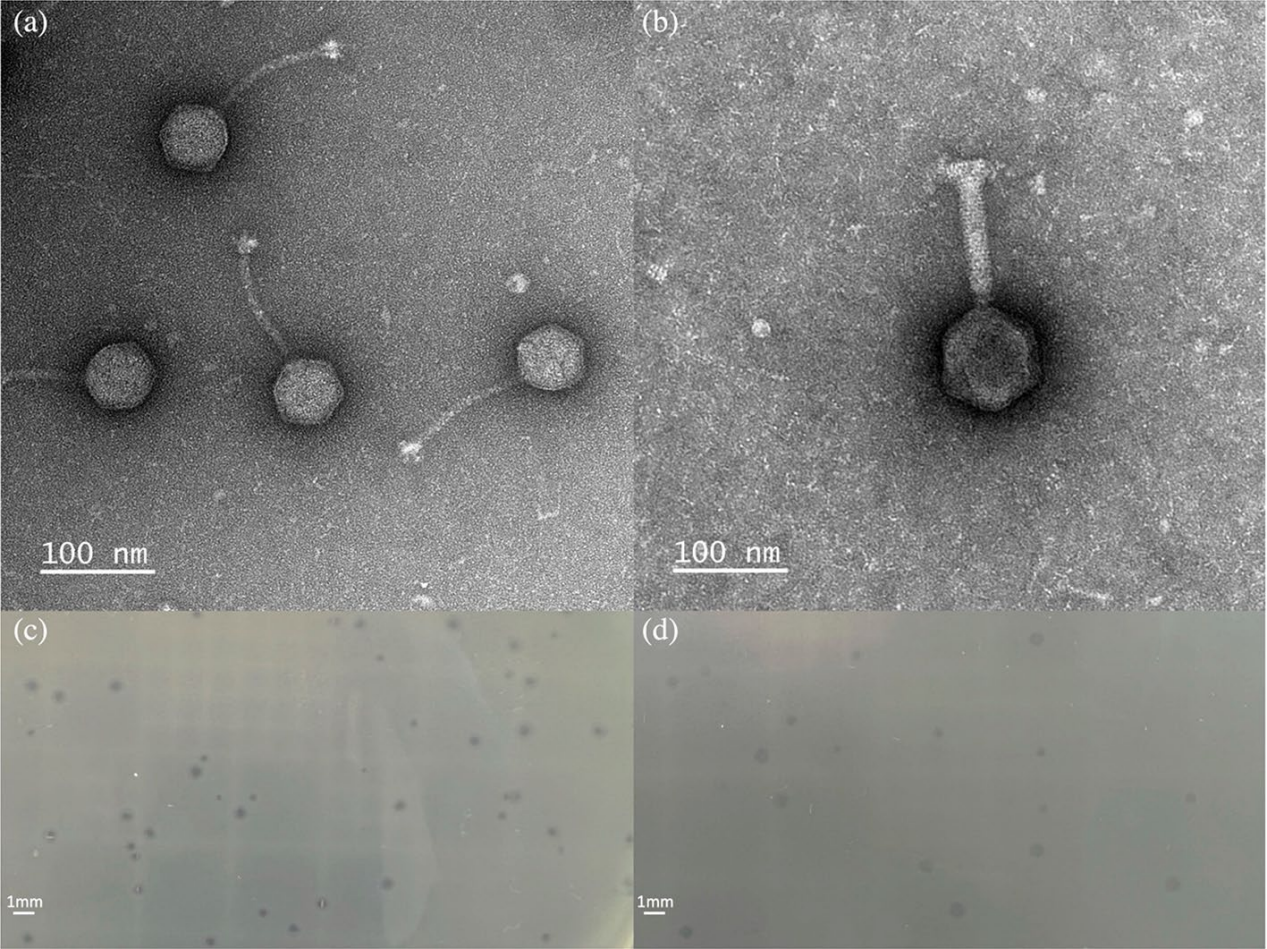
Transmission electron microscopy image of S4lw (**a**) and D5lw (**b**). The morphology of phage S4lw (**a**) has a capsid (64.5 ± 0.5 nm in diameter) and a long non-contractile tail (132.3 ± 0.5 nm in length), belonging to the *Guernseyvirinae family*. Phage D5lw (**b**) has a morphology containing a capsid (96.7 ± 0.5 nm in diameter) and a long contractile tail (135.5 ± 0.5 nm in length), belonging to the *Ackermannviridae* family. The plaque morphologies of S4lw (**c**) and D5lw (**d**) are shown on a plaque-assay plate against *Salmonella* Typhimurium ATCC14028; both phages produced clear plaques with an average size of 0.523 ± 0.01 mm and 0.517 ± 0.02 mm in diameter, respectively. A distinct halo around each lysis zone caused by phage S4lw was observed on the plates and indicated with a white arrow (**c**).

Phage S4lw had a capsid with approximately 64.5 ± 0.5 nm in diameter and a long non-contractile tail of 132.3 ± 0.5 nm in length (Fig. 4a). Phage D5lw had a capsid of approximately 96.7 ± 0.5 nm in diameter and a long contractile tail of 135.5 ± 0.5 nm in length (Fig. 4b). Both phages produced clear plaques on plaque-assay plates, indicating their lytic capability against *Salmonella* Typhimurium (Fig. 4c and d). Moreover, a distinct halo surrounding each lysis zone caused by phage S4lw was observed on the plate (Fig. 4c).

**Figure 5:**
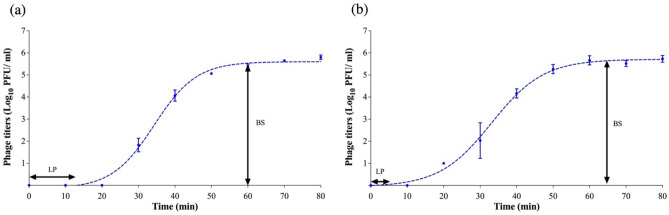
One-step growth curve of the phage S4lw (**a**) and D5lw (**b**) using *Salmonella* Typhimurium ATCC14028. The growth parameters of the phage indicate a latent period (LP) and an average burst size (BS) of each phage. The error bars present the standard error of the mean for each time point of the one-step growth curve.

In addition, the one-step growth curve was conducted against *Salmonella* Typhimurium ATCC14028 for both phages to evaluate their growth factors. The results showed a complete lytic cycle for phage S4lw and D5lw was 65 min and 60 min, respectively (Fig. 5). Phage S4 had a latent period of 12 min with a burst size of 41 PFU per infected cell (Fig. 5a), while Phage D5lw performed a relatively shorter latent period of 4 min with a burst size of 37 per infected cell (Fig. 5b).

### Antimicrobial activity of two phages

**Figure 6:**
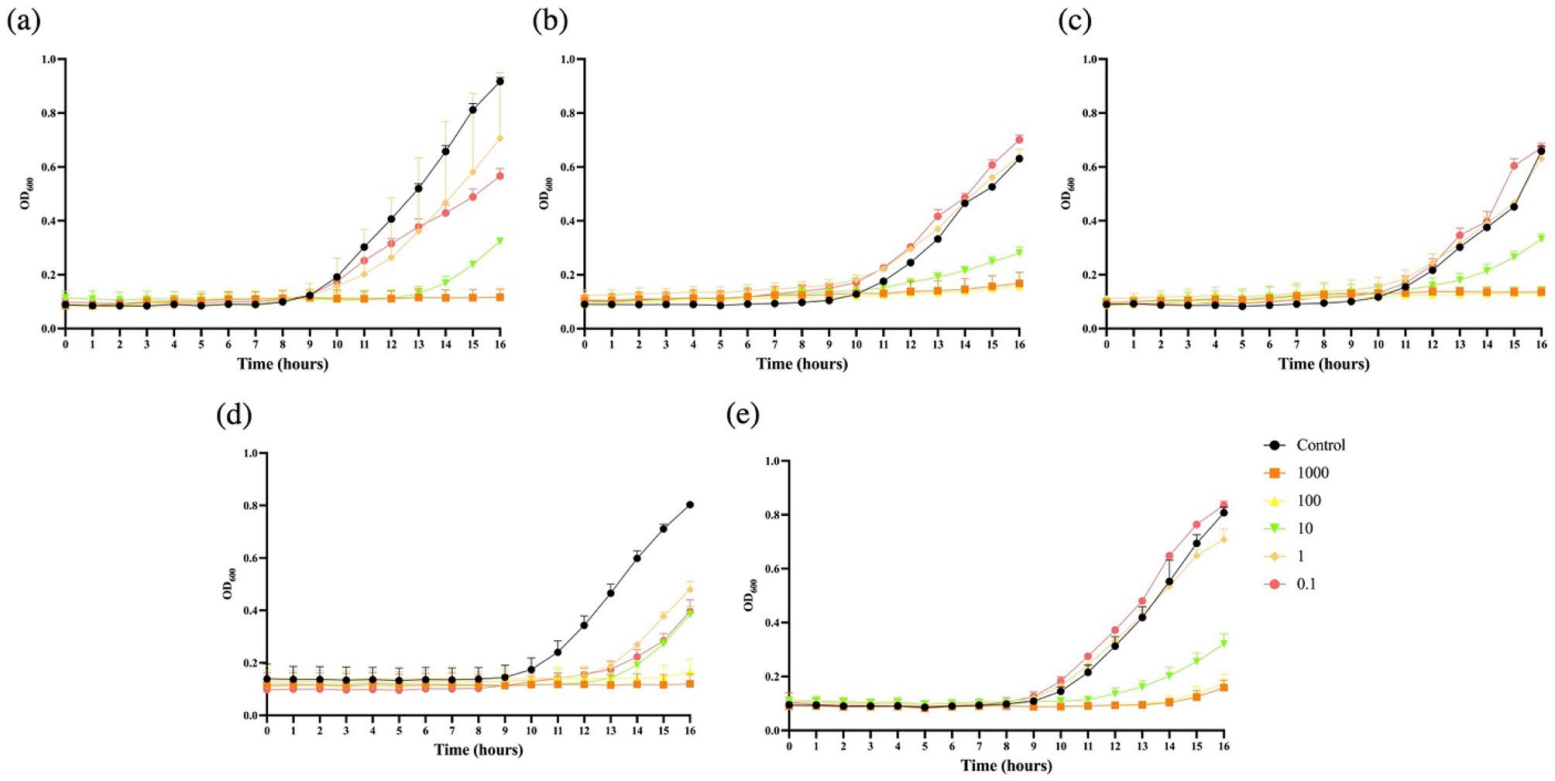
Bacterial challenge assay of a *Salmonella* phage cocktail (S4lw + D5lw) against (**a**) *Salmonella* Typhimurium ATCC14028, (**b**) *Salmonella* Heidelburg 45955, (**c**) *Salmonella* Saintpaul 39, (**d**) *Salmonella* Enteritidis PT-30, and (**e**) *Salmonella* Enteritidis H3527 with different MOIs. The OD_600_ was measured every hour for 16 h at 25 °C. The control contained bacterial culture only. The treatment groups are the mixture of bacterial culture and the phage cocktail with different MOIs of 0.1, 1, 10, 100 and 1000. Each color point is an average of three repeats, with standard error bars.

Each *Salmonella* phage was able to infect a broad spectrum of *Salmonella* serovars, including *S.* Typhimurium, *S.* Enteritidis, *S.* Heidelburg, and *S.* Saintaual (Table 1). The bacterial challenge assay indicated that the two-phage cocktail (D5lw + S4lw) had the highest antimicrobial activity against bacterial host ATCC14028 than the individual phage S4lw and D5lw (data not shown). Therefore, the two-phage cocktail against different *Salmonella* strains at MOIs of 0.1, 1, 10, 100, and 1000 was further determined for biocontrol potential. The results showed that the phage cocktail with the MOI of 100 or 1000 completely inhibited each of the selected *Salmonella* strains for at least 14 h at 25 °C (Fig. 6). Moreover, *S.* Typhimurium ATCC14028, *S.* Enteritidis PT-30, and *S.* Saintpaul 39 were completely inhibited by the phage cocktail with the MOIs of 100 and 1000 for 16 h (Fig. 6a, c and d). In addition, the occurrence of lysis from without for each phage treatment group was determined. The results indicated that no lysis from without was observed on all five tested *Salmonella* strains treated with phage cocktail at the MOIs of 100 and 1,000 (data not shown), demonstrating the strong antimicrobial activities of our phage cocktail.

**Table 1:** Host range of phage S4lw and D5lw against various *Salmonella enterica* serovars and *E. coli* strains.

Bacterial host	Strains ID	Phage S4lw	Phage D5lw
*Salmonella*	*Salmonella* Typhimurium (ATCC14028) (host strain)	+ + *	+ +
	*Salmonella* Typhimurium (6962)	−	−
	*Salmonella* Enteritidis (PT-30)	+ +	+ +
	*Salmonella* Enteritidis (H3527)	+ +	+ +
	*Salmonella* Saintpaul (39)	+	+ +
	*Salmonella* Heidelburg (45955)	+	+ +
	*Salmonella* Montevideo (S1)	−	−
	*Salmonella* Newport (H1073)	−	−
	*Salmonella* Agona (W0081)	−	−
	*Salmonella* Anatum (W0082)	−	−
	*Salmonella* Infantis (ATCC BAA-1675)	−	−
	*Salmonella* Infantis (RM2480)	−	−
*Escherichia coli*	Generic *E. coli* (ATCC15597)	−	−
	Generic *E. coli* (ATCC13706)	−	−
	*E. coli* O26 (RM8426)	−	−
	*E. coli* O45:H- (RM10729)	−	−
	*E. coli* O45:H16 (RM11911)	−	−
	*E. coli* O45:H2 (SJ7)	−	−
	*E. coli* O103:H11 (RM8385)	−	−
	*E. coli* O111 (RM9975)	−	−
	*E. coli* O121:H19 (96–1585)	−	−
	*E. coli* O121 (RM8352)	−	−
	*E. coli* O145:H28 (RM13514)	−	−
	*E. coli* O145:H28 (RM12581)	−	−
	*E. coli* O157:H7 (RM18419)	−	−
	*E. coli* O157:H7 (RM9995)	−	−
	*E. coli* O157:H7 (ATCC43888)	−	−
	*E. coli* O157:H7 (RM19259)	−	−
	*E. coli* O113:H2 (RM9245)	−	−

*Indicates the degree of lysis using a cross mark, “ +  + ” indicates complete lysis, “ + ” indicates weak lysis, and “−” indicates no lysis; the degree of lysis was present as a comparison of the phage titer (10^6^–10^9^ PFU/ml) of the test strain and the primary strain used for phage isolation (ATCC14028).

**Figure 7:**
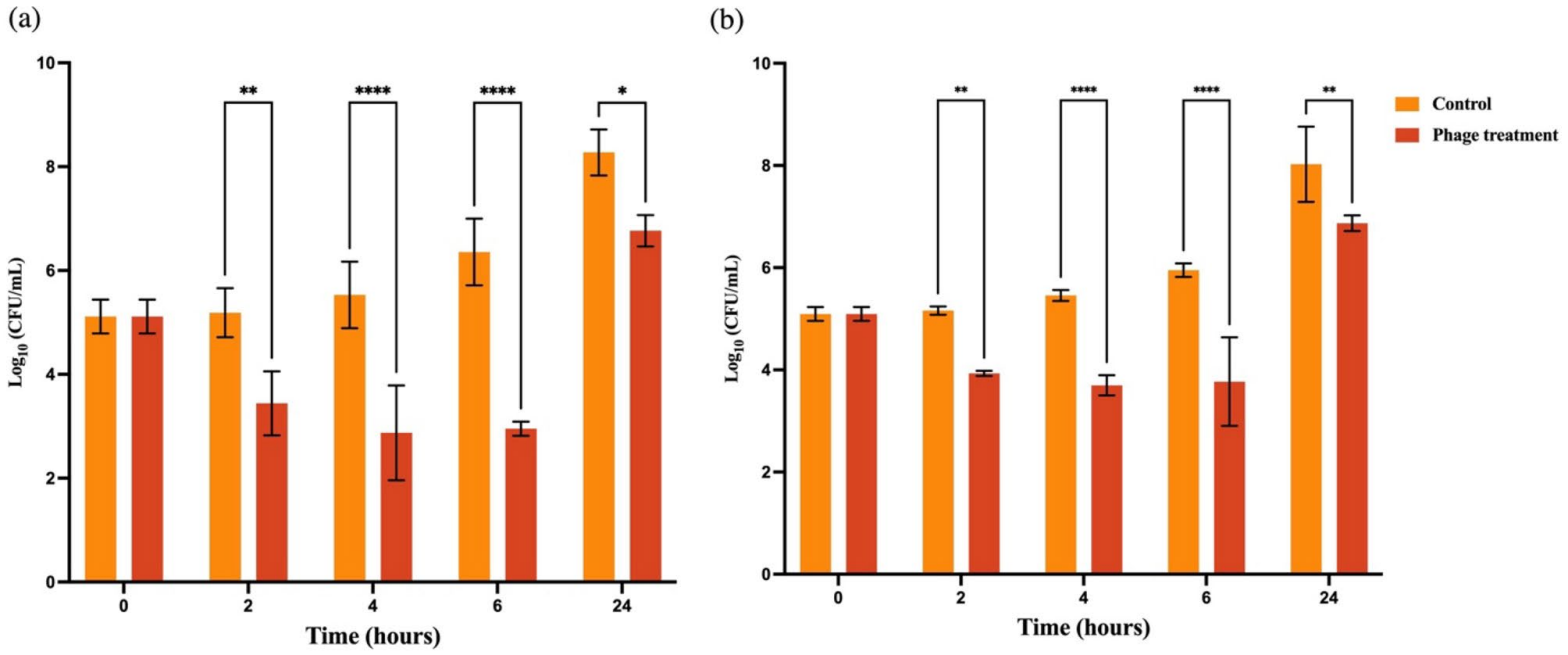
Antimicrobial activities of a two-phage cocktail (S4lw + D5lw) against *Salmonella* Typhimurium ATCC14028 (**a**) and a five-strain bacterial panel (**b**) at an MOI of 100 in TSB at 25 °C for 24 h. The control group contained bacterial culture only. The phage treatment group contained bacterial culture treated with the phage cocktail. Each column represents an average of three repeats, with standard error bars. Data of each time point (0, 2, 4, 6, 24 h) were analyzed separately, and an asterisk indicates significant differences between the control and phage treatment groups. *p < 0.05, **p < 0.01, ***p < 0.001, ****p < 0.0001.

Additionally, during the treatment of *S.* Typhimurium (ATCC14028) by the phage cocktail, the results revealed that the phages significantly reduced the bacterial concentration and attained the highest reduction of 3.4 log after 6-h treatment in comparison to the control (Fig. 7a). A similar result was observed for the phage cocktail against a bacterial panel of five- *Salmonella* strains (Fig. 7b). The phage treatment group had significantly lower bacterial levels than the control group, causing a 1.7–2.2-log reduction after 4 to 6 h of the phage treatment.

### Phage encapsulation and in vitro stability test in acid environments

To enhance our phage stability against *Salmonella* spp. under various acid stresses, the phage cocktail was encapsulated in sodium alginate with different sizes of beads (Supplementary Table S1). The results showed that a 200-µm nozzle size produced the smallest phage-encapsulated beads, with an average of 0.219 mm and the highest encapsulation efficacy of 4.4%, among the 300-, 450-, 750-, and 1000-µm nozzle sizes. Therefore, the encapsulated phages from the 200-µm nozzle size were used for the downstream analysis.

**Figure 8:**
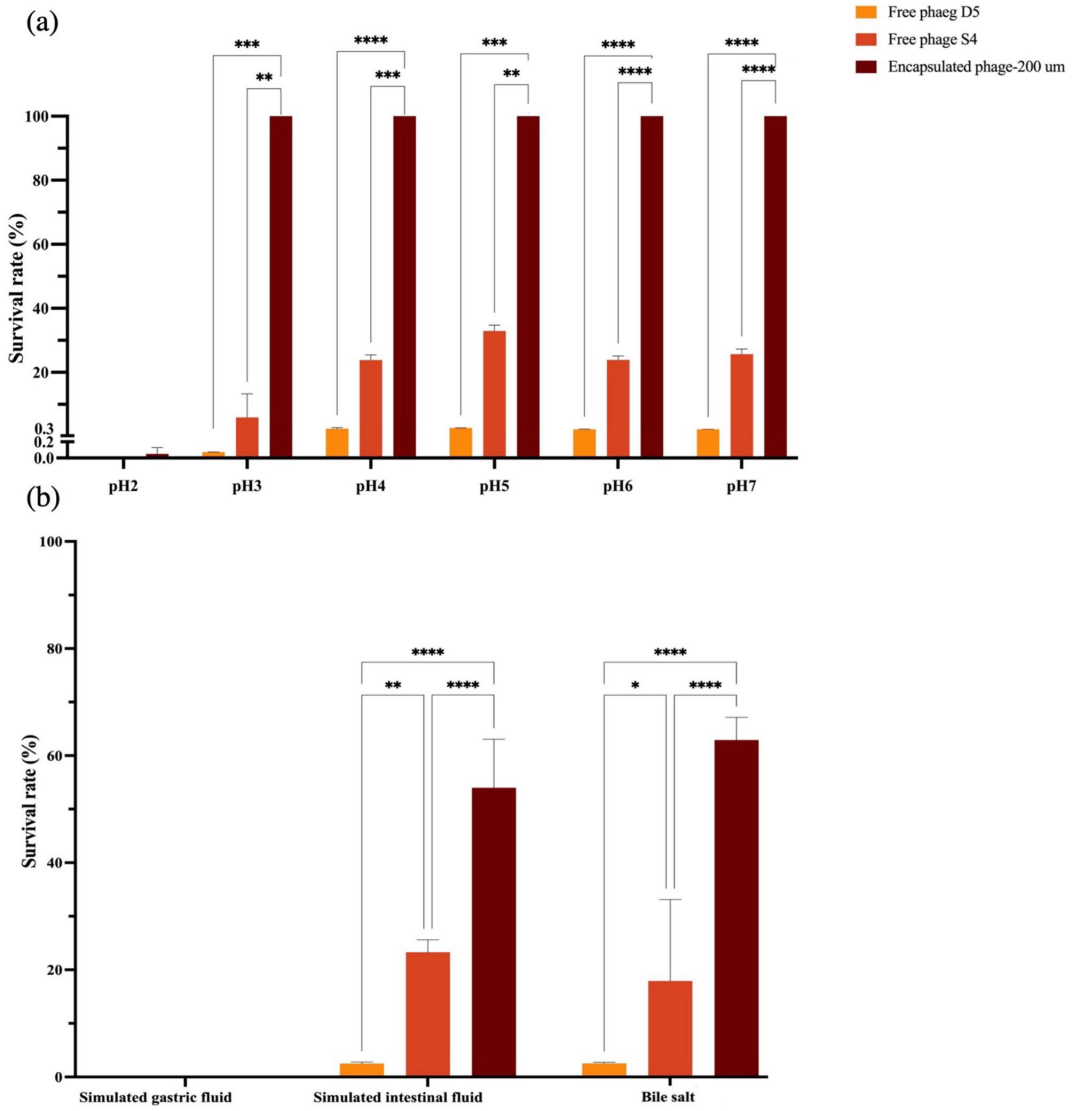
pH test of free phages S4lw, D5lw, and encapsulated phage cocktail produced by a 200-µm nozzle size. (**a**) The phages were added to the SM buffer with different pH levels (pH2, pH3, pH4, pH5, pH6, and pH7) at 42 °C for one hour before neutralization and the survival phage determination. (**b**) The phages were added to the different gastrointestinal tract (GIT)-related conditions (simulated gastric fluid (SGF), bile salt, and simulated intestinal fluid (SIF)) at 42 °C for one hour before neutralization and the survival phage determination. Each column represents an average of three repeats, with standard error bars. Data of each pH level were analyzed separately, and the asterisk indicates significant differences among phages S4lw, D5lw, and encapsulated phage cocktail. *p < 0.05, **p < 0.01, ***p < 0.001, ****p < 0.0001.

pH ranging from 2 to 7 was chosen for the pH stability test on free phages and the encapsulated phage cocktail at 42 °C, mimicking the gastrointestinal tract (GIT) temperature of chickens (Fig. 8a). The result disclosed both free phages S4lw and D5lw were below the detection level at pH 2; however, the encapsulated phages with around 2.58 log10 PFU/mL (0.05%) were recovered after the treatment at pH 2 for one hour. In addition, around 5% free phage S4lw (around 6.67 log_10_ PFU/mL) was detected at pH 3 and 20–30% of the phage survived during pH 4–7. However, free phage D5lw had an average of 0.07% survival at pH 3 and 20% survival rate from pH 4 to 7 under the same condition. Additionally, most encapsulated phages maintained a similar titer before and after treatment from pH 3 to 7 at 42 °C.

Both free and encapsulated phages were used for the stability test in the simulated GIT fluids at 42 °C (Fig. 8b). The result showed that both free and encapsulated phages were inactivated in the simulated gastric fluid (SGF) at pH 2.4. Free phages S4lw and D5lw demonstrated a low survival rate of 17.9% and 2.5% in a simulated bile salt solution, respectively, whereas 64% of the encapsulated phages were detected. Similar results were observed in the simulated intestinal fluid (SIF) for free phages (23.27% for phage S4lw and 2% for phage D5lw) and encapsulated phage cocktail (54%). These results indicated that encapsulation rendered additional protection for free phages to sustain the gastrointestinal stresses.

## Discussion

In this study, two phages—S4lw and D5lw—were isolated from environmental samples and characterized as the potential natural predators against various pathogenic *Salmonella* spp. As the most abundant biological entities in the biosphere, a vast number of phages remain to be explored. The taxonomy guideline by the International Committee on Taxonomy of Virus (ICTV) indicates that two phages can be assigned to the same species if their genome identical (coverage x identity) exceeds 95% at the nucleotide level over the full genome length by use of genomic tools, such as BLASTn. The BLASTn result in the current study showed that phage S4lw shared the highest nucleotide sequence similarity (84.17%) to *Salmonella* phage SGPC (OK169616) (coverage of 91% and identity of 92.50%), while phage D5lw shared the highest nucleotide identical of 88.96% (coverage of 91% and identity of 97.76%) with *Salmonella* phage moki (NC_049506). The comparative genomics also confirmed that phages S4lw and D5lw were not classified to any known species under the *Jerseyvirus* genus and verified species under Genus *Kuttervirus,* respectively (data not shown). As a result, phages S4lw and D5lw are likely proposed as two new species under the respective genera of *Jerseyvirus* and *Kuttervirus*, contributing to the diversity of the phage pool.

Phage stability is a critical factor ensuring the efficiency of phage-based treatment or phage therapy after administration. Temperature and pH are two of the common environmental factors that influence phage stability. To evaluate the effectiveness of our phage cocktail against *Salmonella* in a chicken GIT environment, low pH values with 42 °C were used to test phage stability in this study. The acidic test results indicated that free phage D5lw was not detected at pH 2 and 3; thus, a protection method was necessary to enhance the stability for oral delivery of the phage. Several hydrogel-based materials, including whey protein isolate (WIP), Eudragit S100, and chitosan, have been applied by encapsulation for phage oral delivery^13,14^. In the current study, sodium alginate was used for phage encapsulation due to the industry's low production cost of potetial application. In contrast to free phages, the stability results indicated that the encapsulation process did improve the phage survival rate at pH 3–7 and in the simulated intestinal system. However, the results showed that both free and encapsulated phages were completely inactivated in the SGF at pH 2.4 after 1-h incubation at 42 °C. Our finding was consistent with a previous study that free phages were rapidly inactivated in 20 min at pH 2.0, and encapsulated phages also reached a complete inactivation after 60-min treatment at pH 2.0^15^. Chicken gizzard has the lowest pH environment around pH 2–3 compared to other GIT locations^16^. To overcome the low-pH challenge in chicken gizzards, where food usually stays for 45–60 min^16,17^, the mixture of encapsulated phages and feed may improve the phage survival rate and allow some phages to be delivered to the intestines. A previous study incorporated encapsulated phages in animal feed pellets to test the stability of encapsulated phages after extruding and acid exposure^18^. Their results indicated that alginate-only capsules had no phage loss following extrusion during pellet production and showed a better phage survival rate after acid exposure than the control group. These findings provide a solution regarding oral delivery of phages by adding the encapsulated phages into animal feed. More studies regarding the stability of our encapsulated phages during the pellet production process and acid exposure are necessary.

Overall, two new lytic phages, S4lw and D5lw, were isolated and characterized for controlling *Salmonella* in different conditions. These two phages have no harmful genes in their genomes and are safe for phage application. Both phages have a broad lysis spectrum and can render strong lytic activities against various *Salmonella* serovars. Additionally, alginate was used to encapsulate the two-phage cocktail and provided extra protection for the phages to sustain acidic stress in simulated gut environments. The findings of this study demonstrate that phages S4lw and D5lw are promising biocontrol agents and could be further developed for oral phage therapy to combat *Salmonella* pathogens in poultry gut environments. Future studies regarding the treatment effectiveness and safety of our *Salmonella* phage cocktail against *Salmonella* pathogens in poultry via oral delivery application are required.

## Methods

### Bacteria culture

A collection of the *Salmonella* enterica strains were obtained from the Produce Safety and Microbiology (PSM) Research Unit at the U.S. Department of Agriculture (USDA), Agricultural Research Service (ARS), Western Regional Research Center (WRRC), Albany, CA, United States for this study (Table 1). *Salmonella* Typhimurium ATCC14028 was used for phage propagation and quantification. Fresh bacterial culture was prepared by inoculating 10 ml TSB with 1 μl loopful of each strain for overnight incubation at 37 °C before use.

### Phage isolation

*Salmonella* phages S4lw and D5lw were isolated from sewage water collected from the University of California, Davis sewage plant using the bacterial host of *Salmonella* Typhimurium ATCC14028. Two phages—S4lw and D5lw—were purified using the single-plaque purification method and further propagated with overnight ATCC14028 culture and 10 mM CaCl_2_ in 40 mL of tryptic soy broth (TSB; Difco, Becton, Dickinson, Sparks, MD USA) at 37 °C for 18 h. The enriched phages were centrifuged at 8000 × *g* for 10 min at 4 °C and filtered through a 0.22-μm filter membrane to remove bacterial debris. The obtained phage lysates were concentrated via a 50 kDa cutoff Amicon Ultra-15 Centrifugal Filter Unit (Merck Millipore, Billerica, MA, USA) and purified by CsCl gradient ultracentrifugation as previously described^19^.

### Whole genome sequencing and genomic analysis of phage S4lw and D5lw

The DNA of phage S4lw and D5lw was extracted using a Norgen Biotek phage DNA extraction kit (Thorold, ON, Canada) following the manufacturer’s instructions. The whole genome sequencing was conducted as previously described^20^. Briefly, the DNA library of two phages was constructed using a TruSeq Nano DNA library prep kit (Illumina, San Diego, CA) with the 500 bp sequence length and then sequenced on an Illumina MiSeq sequencer (Illumina, San Diego, CA, USA) based on the manufacturer’s instructions. A total of 12,529,574 and 6,694,250 sequence reads were generated for phages S4lw and D5lw, respectively. The phage genome assembly and annotation were processed as previously described^21^. First, the high-quality reads were obtained using FASTQC and Trimmomatic with the setting of Q30 and then subjected to the de novo assembly using Megahit (version 1.2.9). Genome annotation was performed using RAST (Version 2.0) and Prokka (Version 1.14.5) with the default parameters. The final annotation of two phages was confirmed by manual correction using Geneious (version 2023.02, Biomatters, New Zealand) based on BLASTp results against the Uniprot database, with the nucleotide similarity above 90%. The screening of tRNA, virulence genes, and antibiotic resistance genes in the phage genomes was performed via tRNAscan-SE v.2.0^22^, VirulenceFinder v2.0 (https://cge.cbs.dtu.dk/services/VirulenceFinder/; accessed on 02/01/2023)^23^, and ResFinder v4.1 (https://cge.cbs.dtu.dk/services/ResFinder/; accessed on 02/01/2023)^24^, respectively.

The complete genomes of phages S4lw and D5lw were subjected to taxonomic classification and phylogenetic analysis with other closely related reference phage genomes using BLASTn and ViPTree version 3.4^25^, respectively. The functional genes related to phage replication, bacterial host recognition, and host cell lysis were extracted and subsequently compared with the counterparts of the reference phage genomes via MEGA11 with the ClustalW algorithm and Maximum likelihood tree^26^.

### Phage morphology

The morphologies of *Salmonella* phages S4lw and D5lw were observed using transmission electron microscopy (TEM) (Tecnai G2 F20 model FEI, USA) as previously described^19^.

### One-step growth curve

One-step growth curve experiments were conducted as previously described with subtle modification^19^. Phage (S4lw or D5lw) was added to the bacterial culture of *S.* Typhimurium ATCC14028, with a multiplicity of infection (MOI) of 0.01, and incubated at 37 °C (10 min) for phage adsorption. After centrifuging at 5000 × *g* for 5 min at 4 °C, the bacterial pellet was washed three times with 2 mL of TSB and subsequently resuspended in 20 mL TSB. The resuspended culture was further 100-fold diluted in 30 mL TSB and incubated at 37 °C with shaking at 90 rpm throughout the entire experiment. Upon culture resuspension, phage-infected bacterial cell counts were determined by mixing 50 μL of the sample (diluted culture) with 100 μL of the overnight culture of *S.* Typhimurium ATCC14028 and 5 mL of molten 50% TSA agar prior to pouring into a pre-poured 15-mL TSA plate. During the incubation, 1 mL of sample was collected at a 10-min interval for phage D4lw and S4lw for a total of 80 min. The sample from each time point was filtered through a sterile 0.22-μm membrane filter for double-layer plaque assay to determine phage latent periods and burst sizes. The one-step growth curve experiment of each phage was performed three times.

### Determination of phage host range

Phages S4lw and D5lw were tested for their host range against nine different serovars of *Salmonella* strains (Table 1) using the spot test assay as previously described^19^. Briefly, 5 μl of each tenfold diluted phage lysate (6–9 log PFU/mL) were spotted on the TSA plates pre-mixed with individual bacterial cultures (around 8 log CFU) from the selected host panel in 5 ml molten 50% TSA on the top layer. The level of clearing on the spotted area was observed to evaluate the phage lysis ability against different strains.

### Antimicrobial activity of phage cocktail against Salmonella

The bacterial challenge assay of a two-phage cocktail (S4lw and D5lw with the ratio of 1:1) against different *Salmonella* strains with different MOIs was determined using a spectrophotometer (Promega, Madison, WI, USA). In brief, 180 μl per well of the diluted bacterial culture at the concentration of 1 × 10^5^ CFU/ml was dispensed to a 96-well plate. Subsequently, an aliquot of 20 μl of phage cocktail per well was added to reach the MOIs of 0.1, 1, 10, 100, and 1000 accordingly. Twenty microliters of SM buffer without phage were added as the control. Both control and phage treatment groups with each MOI had three replications. The optical density at 600 nm (OD_600_) reading was recorded every 5 min for the first 1 h to determine the bacterial lysis without complete phage infection (lysis from without) and every hour for 16 h to access the antimicrobial activity of the phage cocktail at room temperature (25 °C).

In addition, the antimicrobial activities of two phages against either a single bacterial strain of ATCC14028 or a panel of five susceptible *Salmonella* strains (*S.* Typhimurium ATCC14028, *S.* Enteritidis PT-30, *S.* Enteritidis H3527, *S.* Saintpaul 39, *S.* Heidelburg 45955) were determined as previously described^27^. The overnight bacterial culture was diluted in 40 mL of LB broth (Invitrogen, Carlsbad, CA, USA) to reach the final concentration of 1 × 10^5^ CFU/ml. The phage cocktail was added to the bacterial solution with an MOI of 100 as the treatment group, while the control group was added with the same volume of SM buffer. The control and treatment groups were incubated at 25 °C for 24 h. Meanwhile, the bacterial concentration was determined at 0, 2, 4, 6, and 24 h by spread plating on XLD (BD, Franklin Lakes, NJ) with thin TSA overlay (BD, Franklin Lakes, NJ) (Thin Agar Layer Method, TAL)^28^.

### Encapsulation of a Salmonella two-phage cocktail

The encapsulation of a phage cocktail—S4lw and D5lw—was conducted as previously described with minor modification^29^. First, phages S4lw and D5lw at approximately 10^9^ PFU/mL were mixed together at a 1:1 ratio as a phage cocktail. A total of 100 mL of phage cocktail in SM buffer was added with 0.02 M Na_2_CO_3_ (Sigma-Aldrich, Oakville, Ontario, Canada) and around 1.2—1.8% sodium alginate (Buchi, Switzerland), depending on nozzle sizes used for the encapsulation. After alginate was dissolved, the phage-alginate mixture was subject to encapsulation using a Buchi B-390 encapsulator (Buchi, Switzerland) with different nozzle sizes (200, 300, 450, 750, and 1000 μm) accordingly. At the same time, a clean beaker containing 200 mL of 1.8% CaCl_2_ (Sigma-Aldrich, Oakville, Ontario, Canada) with a low-rate spinning stir bar was used to catch and harden the resulting phage-encapsulated beads from the encapsulator. After solidifying, the beads were separated from the solution and washed with SM buffer (three times) to remove any free phages. The beads were stored in the SM buffer at 4 °C for further experiments.

### Efficiency of phage cocktail encapsulation using different nozzle sizes

The encapsulation efficacy of phages was determined by measuring the phage concentration encapsulated within the beads as previously described^29^. A total of 1 mL phage-encapsulated beads was added to 9 ml of microsphere-broken solution (MBS) to dissolve the alginate layer and release the phages. A double-layer plaque assay method was used to determine the initial concentration of phages used for encapsulation and the phages released from the encapsulated beads. The encapsulation efficiency (EE) was calculated as EE% = (phage concentration released from the dissolved microspheres /initial phage concentration used for encapsulation) × 100%.

### Acid test of free and encapsulated phages

The pH stability tests, ranging from pH 2 to pH 7, were conducted on free phages S4lw, D5lw, and the encapsulated phage cocktail as previously described with subtle revision^27^. Briefly, 100 μl of single phage S4lw, D5lw, or encapsulated phage cocktail (around 6 log_10_ PFU/mL) was added to 9.9 ml of SM buffer with the final pH values of 2, 3, 4, 5, 6, and 7. The samples were incubated at 42 °C for 1 h before phage quantification via a double-layer plaque assay. For the encapsulated phage, the acid-treated phage beads were dissolved in MBS to release the phage prior to the double-layer plaque assay to determine the stability.

### Phage stability in simulated gastric fluid, bile salt, and simulated intestinal fluid

The stability of free individual phages and the encapsulated phage cocktail was tested under different simulated GIT conditions as previously described^30^. In detail, SGF consisted 3.2 mg/ml pepsin (Sigma-Aldrich, Oakville, Ontario, Canada) in 0.2% (wt/vol) NaCl at pH 2.4. Bile salt contained 1% or 2% (wt/vol) porcine bile extract (Sigma-Aldrich, Oakville, ON, Canada). Simulated intestinal fluid (SIF) had 10 mg/ml pancreatin (Sigma-Aldrich, Oakville, Ontario, Canada) in 50 mM KH_2_PO_4_ at pH 6.8^30^. An aliquot of 100 μl free or encapsulated phages (around 6 log_10_ PFU/mL) was added to 9.9 mL of the solutions (SGF, bile salt, and SIF) at 42 °C for 1 h to determine the phage stability. After 1-h treatment, the survival phage concentration was obtained using the method described in the pH test section.

### Statistical analysis

Experiments were conducted in three individual repeats. Two-way analysis of variance (ANOVA) with Šídák’s multiple comparisons test was used to evaluate the effects of lysis from without, bacterial log reduction, and phage stability tests at a statistical significance of 5% via GraphPad Prism 10.

### Supplementary Information

Supplementary Table S1.

## Data Availability

The complete genome sequences of *Salmonella* phage S4lw and D5lw have been deposited in GenBank under the accession numbers OQ660438 and OQ660437, respectively.
