# Supplementary material for: Characterization of two novel Salmonella phages having biocontrol potential against Salmonella spp. in gastrointestinal conditions
Source: Sci Rep. 2024 May 29;14:12294. doi: 10.1038/s41598-024-59502-9 (PMC11137056; doi:10.1038/s41598-024-59502-9)
Supplement: Supplementary file 1 — Supplementary Table S1. [file 41598_2024_59502_MOESM1_ESM.pdf]

Supplementary materials

Table S1: Summary table of encapsulated phage characteristics produced by different nozzle sizes.

Nozzle	Bead size	Encapsulation efficiency	Optical micrographs of beads
200 µm	219 ± 21 µm	4.4%	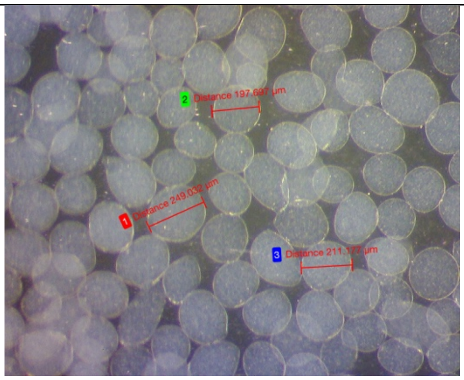
300 µm	284 ± 50 µm	3.3%	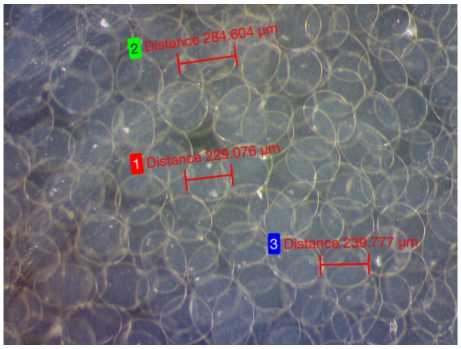
450 µm	303 ± 22 µm	1.1%	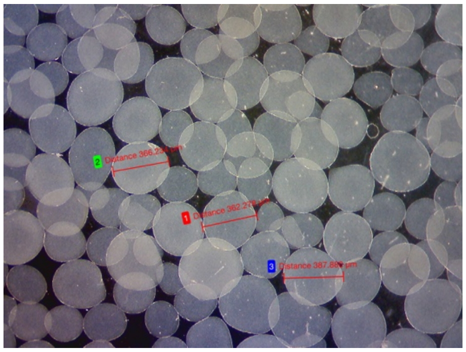

750 µm	574 ± 40µm	0.3%	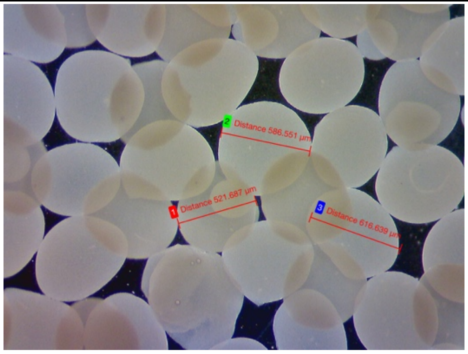
1000 µm	746 ± 37 µm	0.1%	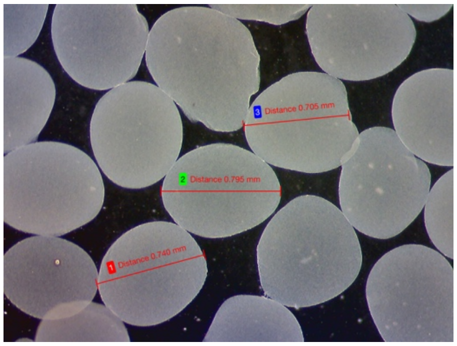
